# Tenement Houses

**Published:** 1875-01

**Authors:** 


TENEMENT HOUSES.— The consequences of ill-health among tenement population, though insidious, are manifest. Among these are, first, a want of sufficient amount of air space, causing the atmosphere respired to be loaded with impurities from the lungs and bodies of those present; second, a want of adequate ventilation to remove these impurities and to admit fresh air sufficient for the support of healthy respiration; third, defective house drainage, allowing the escape from house-drains and waste-pipes of impurities, both of a liquid and gaseous nature; fourth, damp walls, caused by want of previously preparing the ground by proper drainage; fifth, too close proximity of sinks and cesspools, and sixth, improper disposal of house refuse, defiling the yard, the cellar, and the street-gutter with decomposing matter. For the improvement of tenement population the people must be educated in matters pertaining to the care and preservation of health, and this can only be done by extensive voluntary effort.

Their domiciles should be less crowded, should afford more air space, and contain all the facilities for personal and domestic cleanliness and such arrangements as elevate the standard of both physical and moral health.

